# Early Treatment With Curative Dose Low-Molecular-Weight Heparin (LMWH) Versus Plasmapheresis in Severe Acute Pancreatitis in Intensive Care: A Comparative Study

**DOI:** 10.7759/cureus.94994

**Published:** 2025-10-20

**Authors:** Ayadi Yacine, Ouali Mourad, Zeddam Soheib, Houacine Salem

**Affiliations:** 1 Department of Medical Intensive Care, Mustapha Pacha University Hospital, Algiers, DZA

**Keywords:** hypertriglyceridemia, intensive care, low-molecular-weight heparin, plasmapheresis, severe acute pancreatitis

## Abstract

Background: Severe acute pancreatitis (SAP) of hyperlipidemic origin is a serious condition that may require advanced therapeutic interventions (e.g., intravenous fluids, analgesics, insulin infusion, plasmapheresis, nutritional support, and endoscopic/surgical management). While plasmapheresis is used to eliminate circulating triglycerides and inflammatory mediators, low-molecular-weight heparins (LMWH) may offer an alternative through thrombo-inflammatory modulation.

Objective: To compare the efficacy and safety of early therapeutic-dose LMWH versus plasmapheresis in the management of SAP.

Methods: This prospective, observational, single-center study included 100 patients with hypertriglyceridemia-induced SAP. Patients were divided into two groups: 50 received therapeutic-dose LMWH, and 50 underwent plasmapheresis. Primary outcomes included necrosis rate, complication frequency, ICU stay, and mortality. Adverse events related to both interventions were recorded.

Results: LMWH was associated with a lower rate of pancreatic necrosis (8% vs. 44%, p=0.0003), fewer overall complications (10% vs. 60%, p=0.0001), and a shorter ICU stay (6.4 vs. 12.7 days, p<0.001). Mortality was lower in the LMWH group (2% vs. 14%, p=0.03). Procedure-related adverse events were less frequent with LMWH than with plasmapheresis.

Conclusion: In this single-center observational study, therapeutic-dose LMWH was associated with improved clinical outcomes and fewer adverse events compared to plasmapheresis. These findings suggest LMWH may be a safe and effective option for managing hypertriglyceridemia-induced SAP, though randomized controlled trials are needed to confirm these results.

## Introduction

Severe acute pancreatitis (SAP) is a critical inflammatory condition that can rapidly progress to multiple organ failure, with mortality rates reaching up to 30% in severe cases [1]. Among its various etiologies, hypertriglyceridemia-induced SAP represents a unique clinical challenge, characterized by profound systemic inflammation and microvascular complications. Hypertriglyceridemia (HTG) accounts for approximately 1-10% of all pancreatitis cases, with a higher incidence reported during pregnancy compared to the general population [2]. This rise has been attributed to the increasing prevalence of obesity and metabolic syndrome worldwide. According to the National Cholesterol Education Program Adult Treatment Panel III (NCEP-ATP III), hypertriglyceridemia (HTG) is classified as borderline high when triglyceride levels are between 150-199 mg/dL (1.7-2.2 mmol/L), high between 200-499 mg/dL (2.3-5.6 mmol/L), and very high when exceeding 500 mg/dL (≥5.6 mmol/L) [3]. In the context of SAP, levels frequently exceed 10 mmol/L (approximately 8.85 g/L), contributing to a pro-inflammatory state and microcirculatory dysfunction that underlie pancreatic necrosis and systemic complications [4].

Current therapeutic strategies for hyperlipidemic SAP focus on two principal approaches: plasmapheresis and low-molecular-weight heparin (LMWH). Plasmapheresis facilitates the rapid removal of circulating triglycerides and inflammatory mediators but is an invasive and costly intervention associated with potential complications, including hypotension, electrolyte disturbances, and catheter-related infections [5]. LMWH, conversely, offers a less invasive alternative by targeting thrombo-inflammatory pathways and preventing microvascular thrombosis, with additional effects on lipid metabolism and systemic inflammation [6]. However, despite its theoretical advantages, the efficacy of therapeutic-dose LMWH in hyperlipidemic SAP has not been comprehensively evaluated in clinical settings [7].

This study presents a prospective, observational, single-center investigation comparing the efficacy and safety of early treatment with therapeutic-dose LMWH versus plasmapheresis in patients with hyperlipidemic SAP admitted to intensive care. Given these uncertainties, this study aimed to compare the efficacy and safety of therapeutic-dose LMWH with plasmapheresis in patients with hypertriglyceridemia-induced SAP requiring intensive care. We conducted a prospective, observational, single-center study to evaluate outcomes, including pancreatic necrosis, complication rates, ICU stay duration, and mortality.

## Materials and methods

Study design and setting

This prospective, observational, single-center study was conducted in the intensive care unit at Mustapha Pacha University Hospital, Algiers, Algeria, from January 15, 2021, to December 30, 2023. The study aimed to compare the efficacy and safety of therapeutic-dose LMWH versus plasmapheresis in patients with severe acute pancreatitis of hyperlipidemic origin.

Patient enrollment

Enrollment was restricted to patients admitted within the first 24 hours to the intensive care unit (ICU) and who fulfilled at least one severity criterion, including hypocalcemia, lactic acidosis, systemic inflammatory response syndrome (SIRS), organ failure defined as a Sequential Organ Failure Assessment (SOFA) score greater than 2 [8], or an Acute Physiology and Chronic Health Evaluation II (APACHE II) score of 8 or higher [9]. Baseline clinical parameters, laboratory values (triglycerides, CRP, lipase), radiological severity scores (Balthazar CT score and CT Severity Index (CTSI)) [10], and clinical severity indices (SIRS, APACHE II) showed no statistically significant differences between the two groups (p > 0.05).

Inclusion and exclusion criteria

Patients aged 18-70 years presenting with acute pancreatitis and serum triglyceride levels >10 mmol/L within 24 hours of ICU admission were included. Severity criteria included hypocalcemia, lactic acidosis, systemic inflammatory response syndrome (SIRS), organ failure (SOFA score >2), or APACHE II score ≥8.

Exclusion criteria included contraindications to LMWH (e.g., active bleeding, allergy) or plasmapheresis (e.g., hemodynamic instability, severe coagulation disorders), initiation of plasmapheresis >48 hours after admission, and non-hypertriglyceridemic pancreatitis.

Study population

The 100 enrolled patients were divided equally into two treatment groups: (i) LMWH group (n=50), receiving therapeutic-dose LMWH, and (ii) plasmapheresis group (n=50), undergoing plasmapheresis sessions. Baseline demographic and clinical characteristics were comparable between groups.

Treatment protocols

LMWH group: Patients received subcutaneous therapeutic-dose LMWH twice daily, with dosing adjusted for body weight and renal function. Anti-factor Xa levels were monitored to ensure therapeutic anticoagulation.

Plasmapheresis group: Patients underwent one session per day for up to three consecutive days. Replacement fluids included albumin or fresh frozen plasma according to patient-specific needs. Hemodynamic status and procedural parameters were closely monitored.

Outcomes

Primary outcomes: Pancreatic necrosis (assessed via contrast-enhanced CT), frequency of overall complications, ICU length of stay, and all-cause in-hospital mortality.

Secondary outcomes: Procedure-related adverse events, including catheter-related infections, hypotension, electrolyte imbalances, bleeding, and ventilator-associated pneumonia. All outcomes were assessed during ICU stay and at discharge.

Data collection and monitoring

Clinical, laboratory, and radiological data were collected prospectively using standardized case report forms. Demographics, baseline labs, and severity scores (SIRS, APACHE II, SOFA, CTSI) were recorded at admission. Laboratory markers, including triglycerides, CRP, and pancreatic enzymes, were monitored regularly. Procedural details and adverse events were documented in real time.

Statistical analysis

Data were analyzed using the IBM SPSS Statistics for Windows, version 29.0 (IBM Corp., Armonk, NY, USA). Continuous variables were expressed as mean ± SD or median (IQR), and categorical variables as frequencies and percentages. Comparisons were made using Student’s t-test or Mann-Whitney U test for continuous variables, and Chi-square or Fisher’s exact test for categorical variables. A p-value <0.05 was considered statistically significant.

Ethical considerations

The study protocol was reviewed and approved by the Institutional Ethics Committee, Mustapha Pacha Hospital, University of Algiers (Approval No.: H-2025-574, dated 04/02/2020). Written informed consent was obtained from each patient or their legal representative prior to enrollment. All procedures were conducted in accordance with the principles outlined in the Declaration of Helsinki and adhered to applicable national and institutional research guidelines. Patient confidentiality was strictly maintained throughout the study by anonymizing all data and restricting access to authorized research personnel only.

## Results

A total of 100 patients with severe acute pancreatitis of hyperlipidemic origin were included, equally divided between the LMWH group (n=50) and the plasmapheresis group (n=50). Baseline demographic and clinical characteristics were comparable between groups (p>0.05) (Table 1). Respiratory failure was the most frequent organ dysfunction at admission (80%), followed by renal (20%) and cardiovascular (8%) involvement, while 15% of patients presented multiple organ failures. P-values were calculated using Chi-square or Fisher’s exact test for categorical variables, and Student’s t-test or Mann-Whitney U test for continuous variables, as appropriate (Table 1).

**Table 1:** Baseline demographic and clinical characteristics of patients with severe acute pancreatitis of hyperlipidemic origin. LMWH: low-molecular-weight heparin

Variable	Total (N=100)	LMWH group (n=50)	Plasmapheresis group (n=50)	p-value
Age, years (mean ± SD)	52 ± 10	52 ± 9	53 ± 11	>0.05
Sex, female (%)	40 (40%)	20 (40%)	20 (40%)	>0.05
Sex, male (%)	60 (60%)	30 (60%)	30 (60%)	>0.05
Overweight/obese (%)	80 (80%)	40 (80%)	40 (80%)	>0.05
Respiratory failure at admission (%)	80 (80%)	40 (80%)	40 (80%)	>0.05
Renal failure at admission (%)	20 (20%)	10 (20%)	10 (20%)	>0.05
Cardiovascular failure at admission (%)	8 (8%)	4 (8%)	4 (8%)	>0.05
Multiple organ failures (%)	15 (15%)	8 (16%)	7 (14%)	>0.05

Patients treated with LMWH demonstrated significantly better outcomes compared to those undergoing plasmapheresis. The LMWH group had lower rates of pancreatic necrosis, overall complications, and mortality, as well as a shorter ICU stay. In contrast, plasmapheresis was associated with higher procedure-related complications, including catheter-related infections, hypotension, electrolyte imbalances, and ventilator-associated pneumonia. The LMWH group had lower rates of pancreatic necrosis, overall complications, and mortality, as well as a shorter ICU stay (Table 2).

**Table 2:** Comparison of clinical outcomes between the LMWH and plasmapheresis groups. P-values were calculated using the Chi-square test for categorical variables and the Student’s t-test for continuous variables. LMWH: low-molecular-weight heparin

Parameter	LMWH (n=50)	Plasmapheresis (n=50)	P-value
Pancreatic necrosis (%)	8% (4)	44% (22)	0.0003
Overall complications (%)	10% (5)	60% (30)	0.0001
Mortality (%)	2% (1)	14% (7)	0.03
Average ICU stay (days)	6.4 ± 2.2	12.7 ± 3.9	<0.001
Catheter-related infections (%)	–	10% (5)	0.03
Hypotension (%)	–	22% (11)	0.001
Electrolyte imbalance (%)	–	18% (9)	0.004
Ventilator-associated pneumonia (%)	–	10% (5)	0.03

Regarding the evolution of pancreatic lesions, after one week of follow-up, the rate of pancreatic necrosis was significantly lower in the LMWH group (8%, or four patients) compared to the plasmapheresis group (44%, or 22 patients), with a statistically significant difference (p = 0.0003). This difference suggests a protective effect of LMWH on pancreatic microcirculation, probably related to its ability to prevent microthrombosis. Conversely, although plasmapheresis allows a rapid reduction of triglycerides and circulating inflammatory mediators, its effect on pancreatic perfusion seems limited.

Regarding general complications, the LMWH group presented a markedly superior safety profile with only 10% minor complications, without serious events. In contrast, 60% of patients in the plasmapheresis group presented at least one complication: arterial hypotension during sessions in 22% of cases, electrolyte imbalances in 18%, catheter-related infections in 10%, and ventilator-associated pneumonia (VAP) in 10%. This difference is highly significant (p = 0.0001), confirming the better tolerance of anticoagulant treatment with LMWH.

In terms of mortality, no deaths were reported in the LMWH group, compared with seven deaths (14%) in the plasmapheresis group (p = 0.03), occurring mainly in patients with extensive necrosis and multiple organ failures. No hemorrhagic events were observed in either group, confirming the good safety profile of LMWH, even at a therapeutic dose.

Finally, the average length of stay in intensive care was significantly shorter in the LMWH group, with an average of 6.4 ± 2.2 days, compared to 12.7 ± 3.9 days in the plasmapheresis group (p < 0.001). These data confirm that LMWH treatment is not only safer but also more effective, reducing the severity of lesions, complications, mortality, and length of stay in intensive care.

The results of this study align with and extend previous research on the role of anticoagulation in acute pancreatitis, while providing novel insights into direct comparisons with plasmapheresis. Previous investigations have suggested beneficial effects of heparins in pancreatitis, but this study represents one of the first direct comparisons with plasmapheresis in hypertriglyceridemic SAP.

Regarding plasmapheresis, Kyriakidis et al. (2005) previously documented its efficacy in rapidly reducing triglyceride levels in acute hyperlipidemic pancreatitis [11]. However, their study did not compare plasmapheresis to other therapeutic approaches and focused primarily on biochemical rather than clinical outcomes. The current investigation extends these findings by directly comparing clinical outcomes between plasmapheresis and LMWH therapy, revealing the superiority of the latter across multiple domains.

The safety profile of LMWH observed in this study is consistent with the findings of Podda et al. (2024) [12], who also reported the safety of anticoagulation in critically ill patients with severe acute pancreatitis [12]. The absence of hemorrhagic complications in the LMWH group, despite the use of curative dosing, provides further evidence supporting the safety of therapeutic anticoagulation in this context when appropriate patient selection and monitoring are ensured [12].

The superior efficacy of LMWH over plasmapheresis demonstrated in this study challenges the traditional paradigm of rapid triglyceride removal as the primary therapeutic goal in hypertriglyceridemic pancreatitis. The results suggest that addressing the thrombo-inflammatory consequences of hypertriglyceridemia through anticoagulation may be more beneficial than directly removing triglycerides through extracorporeal techniques. This finding aligns with emerging concepts of pancreatitis as a thrombo-inflammatory disorder where microvascular compromise plays a central role in disease progression.

For clinicians, the implementation of LMWH therapy in place of plasmapheresis offers several practical advantages. LMWH is widely available, relatively inexpensive, and does not require specialized equipment or training to administer. This accessibility is particularly important in resource-limited settings where plasmapheresis may not be available or may be prohibitively expensive. The simplified logistics of LMWH administration may also facilitate earlier initiation of therapy, potentially improving outcomes through more timely intervention.

The safety profile of LMWH observed in this study is reassuring for clinical implementation. The absence of hemorrhagic complications, even with curative dosing, suggests that with appropriate patient selection and monitoring, LMWH can be safely administered in SAP. However, clinicians should remain vigilant for bleeding risks, particularly in patients with coexisting coagulopathies or those requiring invasive procedures.

## Discussion

Acute pancreatitis (AP) induced by hypertriglyceridemia (HTG) is increasingly recognized as a clinically significant entity, representing approximately 1-10% of all AP cases and up to 50% of those occurring during pregnancy [13]. HTG is now considered the third most common cause of AP after gallstones and alcohol-related etiologies [14]. This growing prevalence is closely associated with rising obesity rates, poor dietary habits, sedentary lifestyles, and metabolic syndrome, especially in younger populations [15]. The pathophysiology of HTG-induced AP involves the excessive accumulation of triglycerides (>10 mmol/L or >885 mg/dL), leading to the formation of toxic free fatty acids that damage pancreatic acinar cells and contribute to a severe systemic inflammatory response [16].

According to the National Cholesterol Education Program Adult Treatment Panel III (NCEP-ATP III), hypertriglyceridemia (HTG) is classified as borderline high when triglyceride levels are between 150-199 mg/dL (1.7-2.2 mmol/L), high between 200-499 mg/dL (2.3-5.6 mmol/L), and very high when exceeding 500 mg/dL (≥ 5.6 mmol/L) [3]. In the context of acute pancreatitis, patients often present with levels well above 10 mmol/L. These dyslipidemias can be either primary (often due to genetic mutations) or secondary to uncontrolled diabetes, alcohol abuse, hypothyroidism, certain medications, or obesity [17]. Notably, many patients exhibit overlapping primary and secondary causes, making diagnosis and management complex.

The results of our study underscore the superior clinical efficacy and safety profile of LMWH in the management of HTG-induced SAP when compared to plasmapheresis. LMWH significantly reduced the incidence of pancreatic necrosis (8% vs. 44%, p = 0.0003), a finding consistent with prior studies indicating the protective effect of anticoagulants on pancreatic microcirculation [16]. Pancreatic necrosis in SAP is closely linked to microvascular thrombosis and ischemia, both of which LMWH can mitigate by its antithrombotic and anti-inflammatory properties [18-20].

Furthermore, LMWH use was associated with a dramatic reduction in overall complications (10% in LMWH vs. 60% in plasmapheresis, p = 0.0001). The complications observed in the plasmapheresis group, such as hypotension, catheter-related infections, and electrolyte disturbances, are well-documented procedural risks of extracorporeal treatments. In contrast, LMWH offers a less invasive and more physiologically compatible therapeutic strategy, minimizing the risk of iatrogenic complications while promoting vascular stability and improved perfusion in the pancreas.

Our findings also revealed a notable difference in mortality rates between the two groups. Although the number of deaths was relatively small, 14% mortality in the plasmapheresis group compared to only 2% in the LMWH group (p = 0.03) is clinically significant. Mortality in SAP often results from complications such as infected necrosis, multi-organ failure, and SIRS. By reducing pancreatic necrosis and systemic complications, LMWH may indirectly contribute to improved survival outcomes.

Additionally, LMWH therapy led to a significantly shorter ICU stay (6.4 ± 2.2 days vs. 12.7 ± 3.9 days, p < 0.001), highlighting its role in accelerating recovery, reducing healthcare burden, and enhancing patient outcomes. Shorter ICU stays not only benefit the patient but also contribute to more efficient resource allocation in critical care units.

While plasmapheresis remains a valid therapeutic option for the acute reduction of triglyceride levels in extreme HTG, its invasive nature, cost, and associated complications limit its widespread application, especially in resource-limited settings. In contrast, LMWH is widely accessible, cost-effective, and easily administered, making it a practical alternative for first-line treatment in hyperlipidemic SAP.

The significant reduction in ICU length of stay with LMWH therapy has important implications for resource utilization in critical care. In the context of limited ICU bed availability, interventions that reduce length of stay can increase overall ICU capacity and potentially improve access to critical care for other patients. The economic implications are also substantial, as ICU care represents one of the most resource-intensive aspects of hospital expenditure.

For future research, this study highlights the need for larger randomized controlled trials to confirm these findings and further refine therapeutic protocols. Questions regarding optimal LMWH dosing, duration of therapy, and potential combination with other lipid-lowering strategies remain to be addressed. Additionally, an investigation into biomarkers that might identify patients most likely to benefit from LMWH therapy could facilitate more personalized treatment approaches.

Despite the significant findings, this study has several limitations. First, the sample size was relatively small, which may limit the generalizability of the findings to broader populations. Second, as this was a single-center, prospective observational study, the possibility of selection bias and incomplete data cannot be ruled out. Third, some confounding factors, such as dietary habits, genetic predisposition, and concurrent metabolic conditions, were not fully assessed, which may have influenced the observed outcomes. Fourth, imaging interpretation and clinical scoring could have been subject to interobserver variability, as standardized blinded assessment was not performed. Finally, the lack of long-term follow-up data prevented us from evaluating recurrence rates and long-term prognosis. Future multicenter, prospective studies with larger sample sizes are needed to validate our results and provide more definitive conclusions.

This study provides valuable insight into the comparative effectiveness of LMWH versus plasmapheresis in managing SAP of hypertriglyceridemic origin. While the findings strongly favor LMWH in terms of reduced pancreatic necrosis, complications, ICU stay, and mortality, several limitations must be considered. The single-center, observational design limits generalizability and introduces the potential for selection bias and unmeasured confounding. The lack of randomization, small sample size, and exclusion of other SAP etiologies further restrict the scope. Additionally, the absence of standardized assessment for necrosis volume, no evaluation of long-term outcomes or functional recovery, and the lack of cost-effectiveness analysis leave gaps in the clinical applicability.

To strengthen evidence in this area, future research should focus on multicenter randomized controlled trials with larger populations, exploration of optimal LMWH dosing strategies, and evaluation of combination therapies. Long-term studies are needed to assess recurrence, persistent complications, and quality of life outcomes. Moreover, identifying biomarkers predictive of LMWH response and conducting cost-effectiveness comparisons will enhance clinical decision-making.

This study provides robust evidence that therapeutic-dose LMWH was associated with lower rates of pancreatic necrosis, fewer complications, shorter ICU stays, and reduced mortality compared to plasmapheresis in patients with HTG-induced SAP.

## Conclusions

This prospective, observational study suggests that therapeutic-dose low-molecular-weight heparin (LMWH) may offer favorable clinical outcomes compared to plasmapheresis in the management of hyperlipidemic severe acute pancreatitis (SAP). LMWH significantly reduced pancreatic necrosis (from 44% to 8%), complications (from 60% to 10%), ICU stay (halved to 6.4 days), and mortality (from 14% to 2%). These benefits are likely due to LMWH’s role in preventing microvascular thrombosis, reducing inflammation, and improving triglyceride clearance. Importantly, LMWH showed a favorable safety profile with no bleeding complications, unlike plasmapheresis, which was linked to multiple procedure-related issues. Given these findings, early initiation of therapeutic-dose LMWH appears to be a promising approach for eligible patients; however, confirmation through randomized controlled trials is warranted before it can be recommended as first-line therapy. However, the study’s single-center, observational design limits generalizability. Future multicenter randomized trials are essential to validate these results, explore optimal dosing, and identify predictive biomarkers. Nonetheless, LMWH presents a promising, practical, and effective option for treating hyperlipidemic SAP in clinical settings.
